# Treatment targets of cellular senescence and circadian rhythms in ischemic stroke

**DOI:** 10.1097/MD.0000000000043220

**Published:** 2025-07-04

**Authors:** Fukang Zeng, Mengjuan Wang, Menghao He, Zhong Li, Yuxing Zhang

**Affiliations:** a Department of Critical Care Medicine, The Hospital of Xiangxi Tujia and Miao Autonomous Prefecture Ethnic Chinese Medicine, Jishou City, Hunan Province, China; b Department of Neurology, The First Hospital of Hunan University of Chinese Medicine, Yuhua District, Changsha City, Hunan Province, People’s Republic of China; c Hunan University of Chinese Medicine, Changsha City, Hunan Province, China; d Department of Neurology, The University of Texas Health Science Center at Houston, Houston, TX.

**Keywords:** cellular senescence, circadian rhythm, ischemic stroke

## Abstract

Ischemic stroke poses a substantial public health burden due to its high incidence, rendering it an urgent medical concern. Emerging evidence suggests that cellular senescence and circadian rhythms are closely linked to the onset of cerebral infarction and may represent promising therapeutic targets. Accordingly, we retrieved datasets GSE16561 and GSE22255 from the Gene Expression Omnibus and extracted cellular senescence- and circadian rhythm-related genes from the GeneCards database. Through bioinformatics analysis, we identified key targets associated with cellular senescence and circadian rhythm in cerebral infarction, specifically CREBBP, FOS, CDK4, MMP9, PTEN, and HIF1A. Complementing these findings with in vitro experiments, our results demonstrate that the expression of these genes and proteins at these core targets are elevated to varying degrees in an in vitro model of cerebral infarction. These findings provide compelling evidence supporting the pivotal role of cellular senescence- and circadian rhythm-related genes in the pathogenesis of ischemic stroke.

## 1. Introduction

Stroke remains one of the leading causes of death and disability worldwide, particularly in China, where an aging population and unhealthy lifestyles have contributed to a rising incidence. Ischemic stroke (IS), also known as cerebral infarction, is particularly detrimental to both physical and mental health, and imposes a substantial economic burden.^[1]^ In 2020, an estimated 3.4 million new stroke cases and 2.3 million stroke-related deaths occurred among individuals aged 40 years and older in China. IS accounted for approximately 15.5 million cases, representing 86.8% of all stroke events in that year.^[2–4]^ Despite advances in diagnostic and therapeutic strategies, the prognosis of IS remains poor due to its acute onset, high severity, and limited treatment options. Therefore, elucidating the pathogenesis of IS and identifying novel therapeutic targets are essential for improving patient outcomes and mitigating its societal burden.

Cellular senescence refers to a state in which cells undergo a progressive decline in function and structural integrity, ultimately entering a stable cell cycle arrest.^[5]^ It is a natural physiological process that gradually reduces cellular metabolic activity and repair capacity over time. Cellular senescence is typically associated with multiple factors, including genetic, environmental, immunological, and intrinsic cellular mechanisms.^[6–8]^ It is considered one of the major contributing factors to the onset and progression of various age-related diseases.^[9]^ Although the mechanisms underlying cellular senescence are not yet fully understood, current research suggests they are closely associated with intracellular signal transduction, gene expression, metabolic alterations, and other cellular processes.^[10]^ Senescent cells can develop a senescence-associated secretory phenotype (SASP), characterized by the secretion of pro-inflammatory cytokines and other senescence-related factors, which contribute to sterile inflammation and tissue damage, potentially affecting neighboring cells and triggering a fragile cascade response.^[11]^ Aging is a significant risk factor for IS.^[12]^ As aging progresses, DNA damage accumulates, which can lead to endothelial cell dysfunction and senescence, thereby exacerbating vascular aging, promoting atherosclerosis, and increasing the risk of IS.^[13]^ Oxidative stress plays a crucial role in cellular senescence.^[14]^ Oxidative stress can trigger chronic inflammatory responses, accelerate endothelial cell senescence, and negatively impact vascular health, thereby increasing the risk of IS.^[15]^ Additionally, ischemic damage induced by IS can lead to excessive cellular senescence in neural cells, with ischemic stress activating the p16/p21 pathway, resulting in cell cycle arrest and the development of a senescent cell phenotype. Furthermore, cellular senescence can worsen tissue damage following IS. Senescent cells secrete large quantities of inflammatory factors, cytokines, and proteases, which can further exacerbate inflammation, apoptosis, and tissue damage.^[16]^

Circadian rhythms are endogenous cellular oscillations that cycle approximately every 24 hours to regulate cellular metabolism.^[17]^ These rhythms are regulated by a transcription-translation feedback loop involving circadian rhythm genes, which are controlled by the central pacemaker in the suprachiasmatic nucleus (SCN). The molecular foundation of circadian rhythms is provided by circadian rhythm genes. The core genes involved include Circadian locomotor output cycles kaput, Brain and muscle ARNT-like1 (Bmal1), among others.^[18]^ Circadian rhythms function within a highly regulated network of complex clock genes and clock-controlled genes, generating rhythmic oscillations that maintain body homeostasis and influence various physiological and pathological processes. A significant association exists between circadian rhythms and IS. The human biological clock adapts to daily changes by regulating numerous physiological processes and metabolic activities.^[19,20]^ Disruptions in circadian rhythms have been shown to elevate the risk of IS.^[21]^ Sleep, which is intrinsically linked to circadian rhythms, can be negatively impacted by disorders such as chronic sleep deprivation, irregular work hours, and prolonged night shifts, all of which may lead to disruptions in the biological clock.^[22]^ Disruptions in circadian rhythms are closely linked to the onset and progression of IS. Disruptions in these rhythms can lead to sleep disorders, which negatively affect immune function, inflammatory responses, and other critical mechanisms involved in IS. Research has shown that drugs or interventions targeting the regulation of the biological clock may help correct circadian rhythm disorders and improve the prognosis for stroke patients.^[23–25]^

In conclusion, cellular senescence and circadian rhythms are crucial regulatory factors in IS, warranting further investigation into their molecular mechanisms through bioinformatics to identify new therapeutic targets.

## 2. Materials and methods

### 2.1. Data download

The IS datasets GSE16561^[26–28]^ and GSE22255^[29]^ were downloaded from the Gene Expression Omnibus (GEO) database (https://www.ncbi.nlm.nih.gov/geo/). Detailed sample information for these datasets is provided in Table 1. Cellular senescence and circadian rhythm-related genes were retrieved from the GeneCards database (https://www.genecards.org/). After merging and removing duplicates, 169 genes related to cellular senescence and circadian rhythms were identified. Batch correction was applied to the datasets to obtain the integrated GEO dataset, which included 59 IS samples and 44 control samples. Finally, the integrated GEO dataset was standardized, and principal component analysis (PCA) was conducted to verify the effectiveness of the batch effect removal.

**Table 1:** GEO microarray chip information.

	GSE16561	GSE22255
Platform	GPL6883	GPL570
Species	Homo sapiens	Homo sapiens
Tissue	Peripheral blood tissue	Peripheral blood tissue
Samples in IS group	39	20
Samples in control group	24	20
Reference	PMID: 20837969 PMID: 28446746 PMID: 29263821	PMID: 22453632

GEO = Gene Expression Omnibus, IS = ischemic stroke.

### 2.2. Differentially expressed gene analysis

The samples were divided from the integrated GEO datasets into the IS group and the control group. The R package limma was used to perform differential gene expression analysis between the IS group and the control group and generate a volcano plot. To identify IS-related cellular senescence and circadian rhythm-related differentially expressed genes (DEGs), we took the intersection of DEGs obtained from the differential analysis of the integrated GEO dataset and the cellular senescence and circadian rhythm-related genes and generated a Venn diagram.

### 2.3. Gene Ontology (GO) and Kyoto Encyclopedia of Genes and Genomes (KEGG) pathway enrichment analyses

The R package clusterProfiler was used to perform GO and KEGG pathway enrichment analysis on cell senescence and circadian rhythm-related DEGs associated with IS. Screening criteria for the entries are *P* value < .05 and false discovery rate value (*q* value) < 0.25, which are considered statistically significant.

### 2.4. Gene set enrichment analysis (GSEA)

To assess the impact of the expression levels of all genes in the integrated GEO dataset on IS, the relationships between gene expression and the associated biological processes (BP), affected cellular components, and molecular functions were examined using GSEA. The genes were ranked in the integrated GEO dataset based on the logFC value, and then the R package clusterProfiler was used to perform GSEA on all genes in the dataset. The selection criteria are adj.*P* < .05 and false discovery rate (*q* value) < 0.25, with the *P* value correction method being Benjamini–Hochberg.

### 2.5. Protein–protein interaction (PPI) network and Hub Genes screening

A PPI network was constructed related to cellular senescence and circadian rhythm differential expression genes associated with IS. We selected genes interacting with other genes in the PPI network as hub genes for further analysis. We used Cytoscape software to visualize the network and calculated the functional relevance of hub genes using the R package GOSemSim to analyze the functional similarity among them.

### 2.6. Immune infiltration analysis of hub genes using single-sample gene-set enrichment analysis (ssGSEA) algorithm

We marked the types of infiltrating immune cells (activated CD8 T cell, activated dendritic cell, gamma delta T cell, natural killer cell, and regulatory T cell, etc), used ssGSEA analysis to obtain the immune cell infiltration matrix, plotted a group comparison chart to show the expression differences of immune cells between the stroke group and the control group in the integrated GEO dataset, screened for immune cells with significant differences between the 2 groups for subsequent analysis, and drew a heatmap to display. We calculated the correlation between Hub Genes and immune cells based on the Spearman algorithm, retained results with *P* value < .05, and used the R package ggplot2 to draw a correlation bubble chart to show the correlation analysis results of Hub Genes and immune cells.

### 2.7. Validation of the differential expression of Hub Genes and ROC analysis

To further investigate the differences in the expression of Hub Genes between the IS group and the control group within the integrated GEO dataset, a comparison plot of group expression levels was generated based on Hub Gene expression. The R package pROC was employed to generate ROC curves for the Hub Genes and to compute the area under the curve (AUC) of these curves. This evaluation aimed to assess the diagnostic efficacy of Hub Gene expression levels in predicting the occurrence of IS.

### 2.8. Construction of a model of oxygen-glucose deprivation in human brain microvascular endothelial cells

A human brain microvascular endothelial cell oxygen-glucose deprivation (OGD) model was established using human brain microvascular endothelial cells/D3 (CD0587, Shanghai Qida Biotechnology Co., Ltd., Shanghai, China) to simulate IS injury in vitro. Human brain microvascular endothelial cells/D3 cells were placed into a 25T culture flask containing 4 mL of complete medium (89% high glucose DMEM, 10% fetal bovine serum, and 1% penicillin–streptomycin mixture) and incubated at 37 °C in a tri-gas incubator (95% O_2_ and 5% CO_2_). Logarithmic phase cells were used for subsequent experiments. The medium of the model group cells was replaced with glucose-free DMEM, and they were then placed under conditions of 5% CO_2_ and 95% N_2_ for 2.5 hours.^[30]^ The glucose-free medium was discarded to establish the human brain microvascular endothelial cell OGD model, and the morphological changes of the cells were observed using a microscope.

### 2.9. The expression of the Hub Genes is detected by RT-qPCR

To verify the mRNA expression of Hub Genes in an oxygen-glucose deprivation model of human brain microvascular endothelial cells, total RNA was extracted using an RNA extraction kit (SimGen, Hangzhou, China) according to the manufacturer’s instructions. cDNA synthesis was subsequently performed using a reverse transcription kit (Novoprotein, Shanghai, China). RT-qPCR was subsequently conducted using an amplification kit (Bio-Rad, Berkeley). The relative quantitative analysis utilized the 2^−ΔΔCt^ method, with β-actin serving as the reference gene. Primer sequences are detailed in Table 2.

**Table 2:** Sequences of the primers for RT-qPCR.

Name	Forward primer sequence (5′–3′)	Reverse primer sequence (5′–3′)
CREBBP	AAGCAGCACGGAAGAGAGAG	GGTTTTCAAGCACTGCCACT
FOS	GCTGACAGATACACTCCAAG	CCTAGATGATGCCGGAAACA
CDK4	AATGTTGTACGGCTGATGGA	AGAAACTGACGCATTAGATCCT
MMP9	CTGGACAGCCAGACACTAAAG	CTCGCGGCAAGTCTTCAGAG
PTEN	CGGAACTTGCAATCCTCAGT	AGGTTTCCTCTGGTCCTGGT
HIF1A	ACAAGTCACCACAGGACAG	AGGGAGAAAATCAAGTCG
β-actin	CACCCGCGAGTACAACCTTC	CCCATACCCACCATCACACC

### 2.10. The protein expression of the Hub Genes is detected by Western blot

To verify the protein expression of Hub Genes in the human brain microvascular endothelial cell oxygen-glucose deprivation model, control and model group cells were collected, RIPA protein lysis buffer was added, and the cells were lysed on ice to extract total cell protein. A 2 g/L protein system was prepared, followed by gel electrophoresis of the protein samples. The membrane was then transferred to a PVDF membrane, blocked with 5% milk at room temperature for 60 minutes, followed by the addition of anti-CREBBP (22277-1-AP, Proteintech, 1:2000), anti-FOS (66590-1-Ig, Proteintech, 1:4000), anti-CDK4 (11026-1-AP, Proteintech, 1:4000), anti-MMP9 (10375-2-AP, Proteintech, 1:2000), anti-PTEN (60300-1-Ig, Proteintech, 1:4000), anti-HIF1A (20960-1-AP, Proteintech, 1:6000), and anti-β-actin (66009-1-Ig, Proteintech, 1:8000). The membrane was incubated at 4 °C overnight, and the following day, it was incubated with anti-rabbit horseradish peroxidase-conjugated secondary antibodies (SA00001-2, Proteintech, 1:8000), and anti-mouse horseradish peroxidase-conjugated secondary antibodies (SA00001-1, Proteintech, 1:8000) at 37 °C for 60 minutes. The membrane was then developed with the ECL efficient chemiluminescence reagent kit, and quantitative analysis was performed using ImageJ software.

### 2.11. Statistical analysis

All data processing and analysis in the bioinformatics section of this article were conducted using R software, while in vitro experiments were performed using GraphPad Prism 8.0.2 for statistical analysis and plotting. For the comparison of 2 groups of continuous variables, the statistical significance of normally distributed variables was estimated using the independent Student *t* test, while differences between non-normally distributed variables were analyzed using the Mann–Whitney *U* test, also known as the Wilcoxon Rank-Sum Test. For the comparison of 3 or more groups, the Kruskal–Wallis test was used. The results were calculated using Spearman correlation analysis to determine the correlation coefficient between different molecules. All statistical *P* values are two-sided, with *P* value < .05 considered statistically significant.

## 3. Result

### 3.1. Merge of stroke datasets

**Figure 1.**
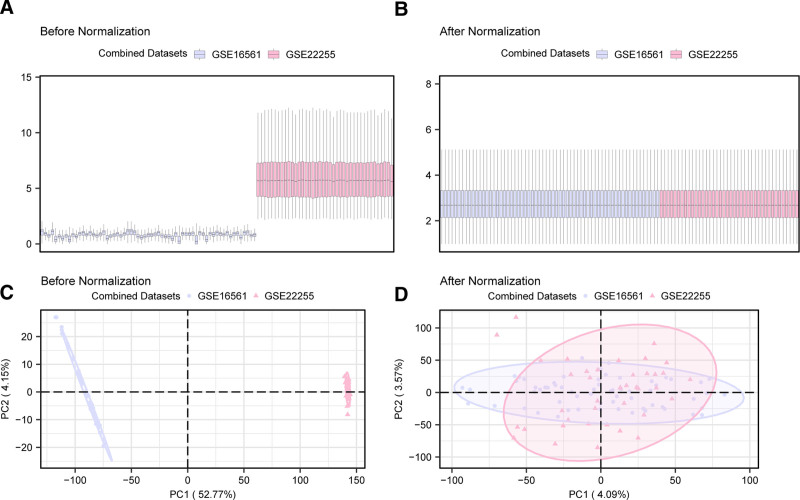
Data de-batching. (A) Box plot of the combined GEO dataset before batch correction. (B) Box plot of the combined GEO dataset after batch correction. (C) PCA plot of the dataset before batch correction. (D) PCA plot of the combined GEO dataset after batch correction. Light purple represents the IS dataset GSE16561, and light red represents the IS dataset GSE22255. GEO = gene expression omnibus, IS = ischemic stroke, PCA = principal component analysis.

**Figure 2.**
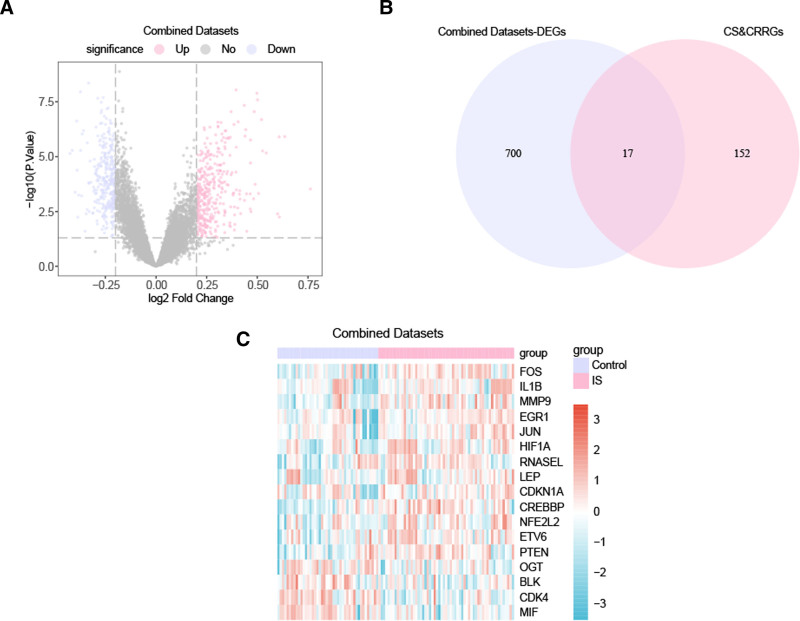
Differential gene expression analysis. (A) Volcano plot of differentially expressed genes between the ischemic stroke (IS) group and control group in the combined GEO datasets. (B) Venn diagram of differentially expressed genes, cell senescence, and circadian rhythm-related genes in the combined GEO datasets. (C) Heatmap of differentially expressed genes related to cell senescence and circadian rhythm in the combined GEO datasets. Light red represents the ischemic stroke (IS) group, light purple represents the control group. In the heatmap, red indicates high expression, and blue indicates low expression. GEO = Gene Expression Omnibus.

The results of the distribution box plot (Fig. 1A and B) and PCA plot (Fig. 2C and D) indicate that the batch effects of samples in the IS dataset were largely eliminated after batch correction.

### 3.2. Genes differentially expressed in IS-related cellular senescence and circadian rhythms

**Figure 3.**
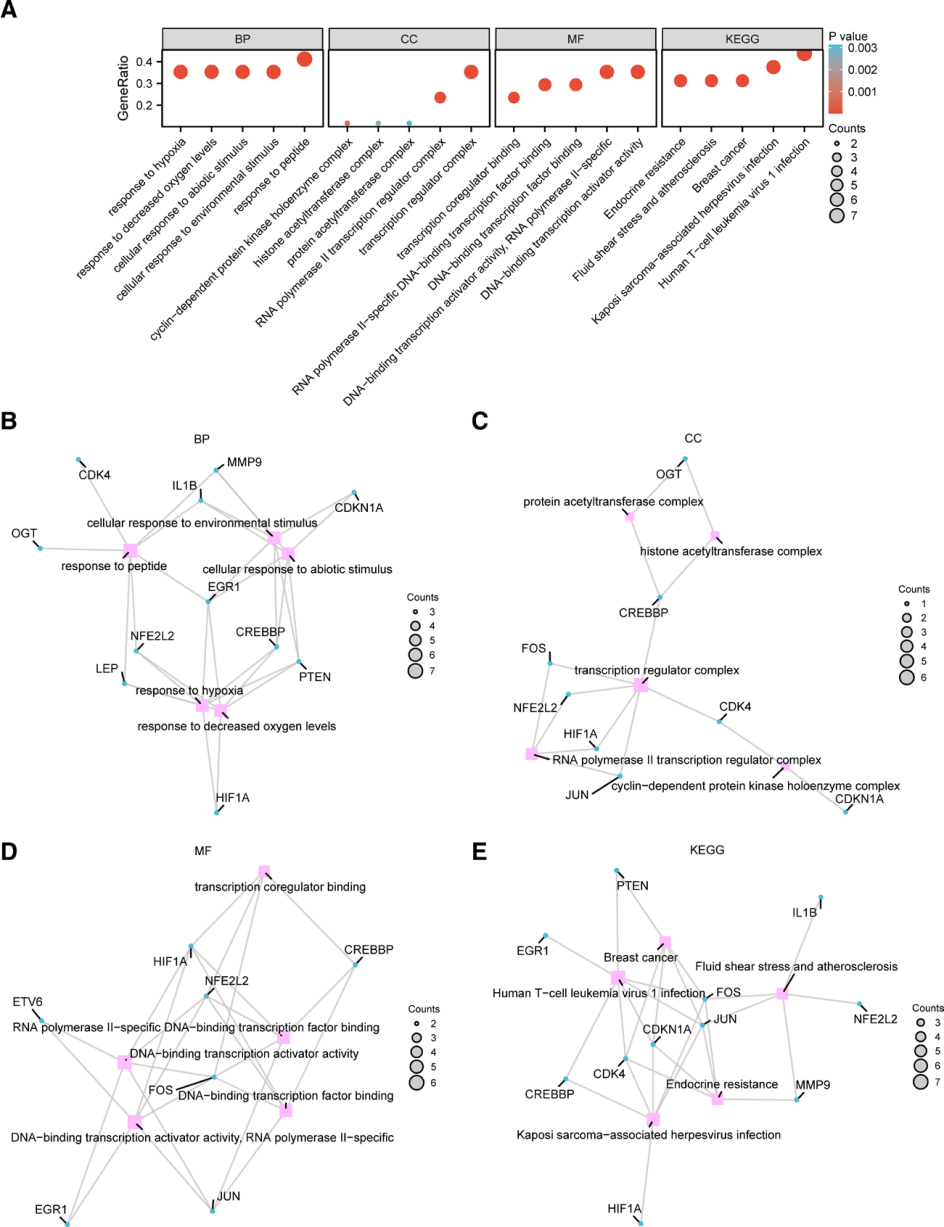
GO and KEGG pathway enrichment analysis of CS & CRRDEGs. (A) The bubble chart of GO and KEGG pathway enrichment analysis results of cell senescence and circadian rhythm-related differentially expressed genes (CS & CRRDEGs) shows biological processes (BP), cellular components (CC), molecular functions (MF), and biological pathways (KEGG). The horizontal axis represents GO terms and KEGG terms. (B–E) The network diagram of GO and KEGG enrichment analysis results of CS&CRRDEGs shows BP (B), CC (C), MF (D), and KEGG (E). Pink nodes represent terms, blue nodes represent molecules, and lines represent the relationships between terms and molecules. In the bubble chart, bubble size represents the number of genes, bubble color represents the *P*-value, with redder colors indicating smaller *P*-values and bluer colors indicating larger *P*-values. The screening criteria for GO and KEGG enrichment analysis were *P*-value < .05 and FDR value (*q* value) < 0.25. FDR = false discovery rate, GO = Gene Ontology, KEGG = Kyoto Encyclopedia of Genes and Genomes.

The combined GEO dataset contains a total of 717 DEGs that meet the threshold of |logFC| > 0.20 and *P* value < .05. Under this threshold, there are 380 upregulated genes (logFC > 0.20 and *P* value < .05) and 337 downregulated genes (logFC < -0.20 and *P* value < .05). A volcano plot illustrating the results of differential analysis for this dataset is shown in Fig. 3A. To identify the DEGs related to cell senescence and circadian rhythm, the intersecting genes between all obtained DEGs and genes related to cell senescence and circadian rhythm were selected, and a Venn diagram was created (Fig. 3B). A total of 17 DEGs related to cell senescence and circadian rhythm in brain infarction were identified: CREBBP, FOS, CDK4, MMP9, MIF, ETV6, EGR1, PTEN, HIF1A, JUN, NFE2L2, OGT, BLK, RNASEL, IL1β, CDKN1A, and LEP. Based on the intersection results, the expression differences of cell senescence and circadian rhythm-related DEGs between different sample groups in the combined GEO dataset were analyzed, and a heat map was created using the R package pheatmap to display the analysis results (Fig. 3C).

### 3.3. Results of GO and KEGG pathway enrichment analyses

The results indicate that the 17 cell senescence and circadian rhythm-related DEGs in IS are primarily enriched in biological processes (BP), including response to peptides, response to hypoxia, response to decreased oxygen levels, cellular response to abiotic stimuli, and cellular response to environmental stimuli. These genes are also involved in cellular components, including the transcription regulator complex, RNA polymerase II transcription regulator complex, cyclin-dependent protein kinase holoenzyme complex, histone acetyltransferase complex, and protein acetyltransferase complex. Regarding molecular functions, they are enriched in DNA-binding transcription activator activity, RNA polymerase II-specific activity, DNA-binding transcription activator activity, RNA polymerase II-specific DNA-binding transcription factor binding, DNA-binding transcription factor binding, and transcription coregulator binding. Additionally, they are enriched in biological pathways (KEGG) related to Human T-cell leukemia virus 1 infection, Kaposi sarcoma-associated herpesvirus infection, endocrine resistance, fluid shear stress and atherosclerosis, and breast cancer. The results of the GO and KEGG enrichment analyses are visualized through a bubble chart (Fig. 3A–E).

### 3.4. Results of GSEA

**Figure 4.**
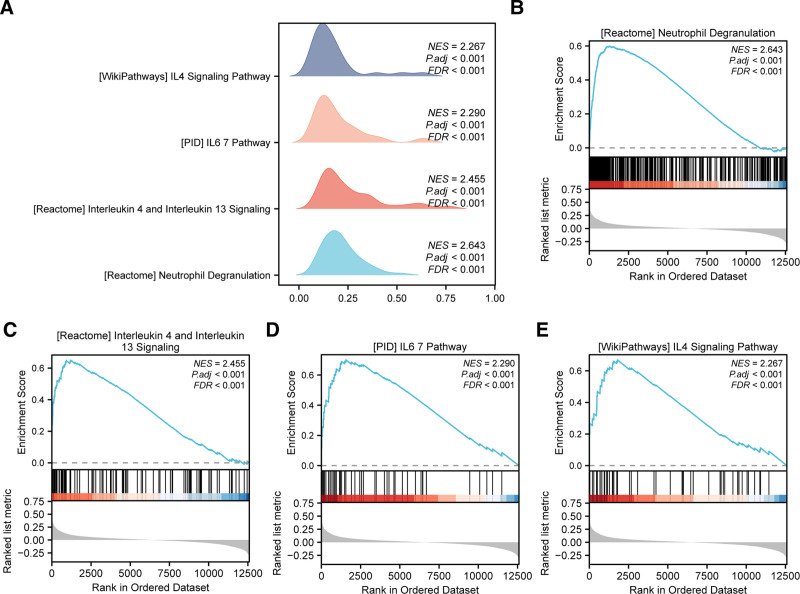
GSEA analysis of ischemic stroke. (A) Ridge plots of 4 biological functions from gene set enrichment analysis of the integrated GEO datasets. (B–E) Gene set enrichment analysis shows all genes are significantly enriched in neutrophil degranulation (B), interleukin 4 and interleukin 13 signaling (C), IL6 7 pathway (D), IL4 signaling pathway (E). The screening criteria for GSEA are adj.*P* < .05 and FDR (*q* value) < 0.25, with *P* value correction method being Benjamini–Hochberg. FDR = false discovery rate, GEO = Gene Expression Omnibus, GSEA = gene set enrichment analysis.

The results showed (Fig. 4A) that all genes in the integrated GEO dataset were significantly enriched in biologically relevant functions and signaling pathways, including neutrophil degranulation (Fig. 4B), interleukin 4 and interleukin 13 signaling (Fig. 4C), IL6/7 pathway (Fig. 4D), and IL4 signaling pathway (Fig. 4E).

### 3.5. Results of PPI network analysis

**Figure 5.**
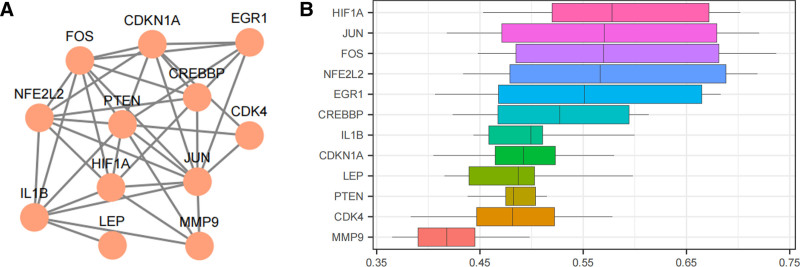
Hub Genes interaction network analysis. (A) Protein–protein interaction network (PPI network). (B) Box plot of functional similarity of Hub Genes. PPI = protein–protein interaction.

The results of the PPI network (Fig. 5A) show that 12 DEGs related to cell aging and circadian rhythms are associated with IS: CREBBP, FOS, CDK4, MMP9, EGR1, PTEN, HIF1A, JUN, NFE2L2, IL1β, CDKN1A, and LEP, which are considered Hub Genes for subsequent analysis. Secondly, based on the functional similarity analysis score, genes that play an important role in the BP of IS are identified (Fig. 5B). The results show that HIF1A plays an important role in IS and is the gene closest to the critical value (cutoff value = 0.60).

### 3.6. Results of immuno-infiltration analysis (ssGSEA)

**Figure 6.**
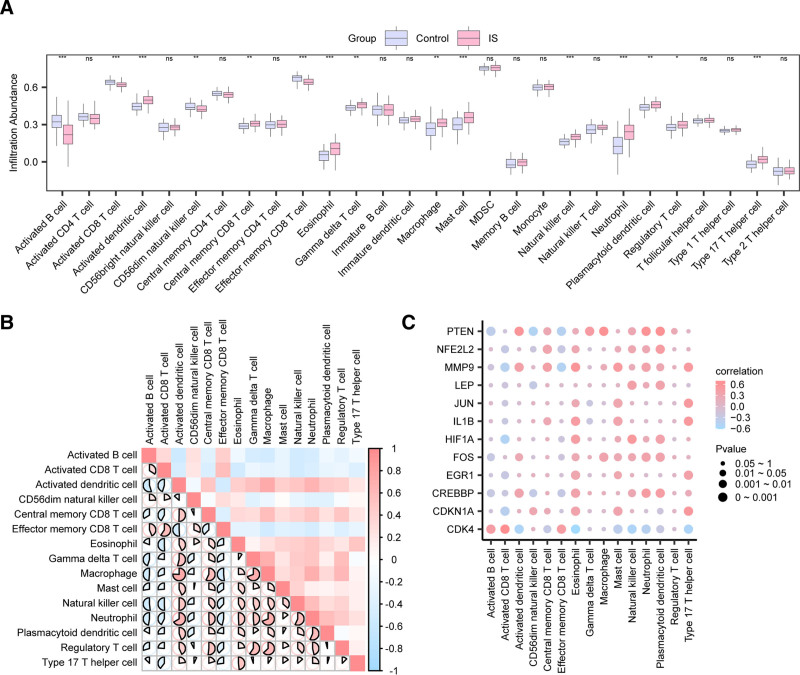
Immunoinfiltration analysis of combined datasets (ssGSEA). (A) Comparison chart of immune cells between IS and control groups in the integrated GEO dataset. (B) Correlation heatmap of immune cell infiltration abundance in the integrated GEO dataset. (C) Correlation bubble chart of Hub Genes with immune cell infiltration abundance in the integrated GEO dataset. ns represents *P* value ≥ .05, not statistically significant; * represents *P* value < .05, statistically significant; ** represents *P* value < .01, highly statistically significant; *** represents *P* value < .001, extremely statistically significant. The absolute value of the correlation coefficient (*R* value) between 0.5 and 0.8 indicates a moderate correlation. In the comparison chart, light purple represents the control group, light red represents the ischemic stroke (IS) group. Red indicates positive correlation, blue indicates negative correlation, and the depth of the color represents the strength of the correlation. GEO = Gene Expression Omnibus, ssGSEA = single-sample gene-set enrichment analysis.

The grouping comparison chart (Fig. 6A) shows that 15 types of immune cells, including activated B cells, activated CD8 T cells, central memory CD8 T cells, effector memory CD8 T cells, gamma delta T cells, regulatory T cells, type 17 T helper cells, activated dendritic cells, CD56dim natural killer cells, eosinophils, macrophages, mast cells, natural killer cells, neutrophils, and plasmacytoid dendritic cells, exhibit statistically significant differences between the IS group and the control group (*P* value < .05). Subsequently, a correlation heatmap was used to display the correlation results of the GEO dataset, integrating the infiltration abundance of these 15 immune cell types in the immune infiltration analysis (Fig. 6B). The results indicate that most immune cells exhibit positive correlations with each other. Furthermore, the correlation between the 12 Hub Genes and the 15 immune cells was analyzed and displayed through a correlation bubble chart (Fig. 6C). The results indicate that among the Hub Genes, CDK4 exhibited the strongest positive correlation with the immune cell activated CD8 T cell (*R* value = 0.70, *P* value < .05), and CDK4 showed the strongest negative correlation with the immune cell Eosinophil (*R* value = ‐0.61, *P* value < .05).

### 3.7. Results of differential expression validation and ROC curve analysis of Hub Genes

**Figure 7.**
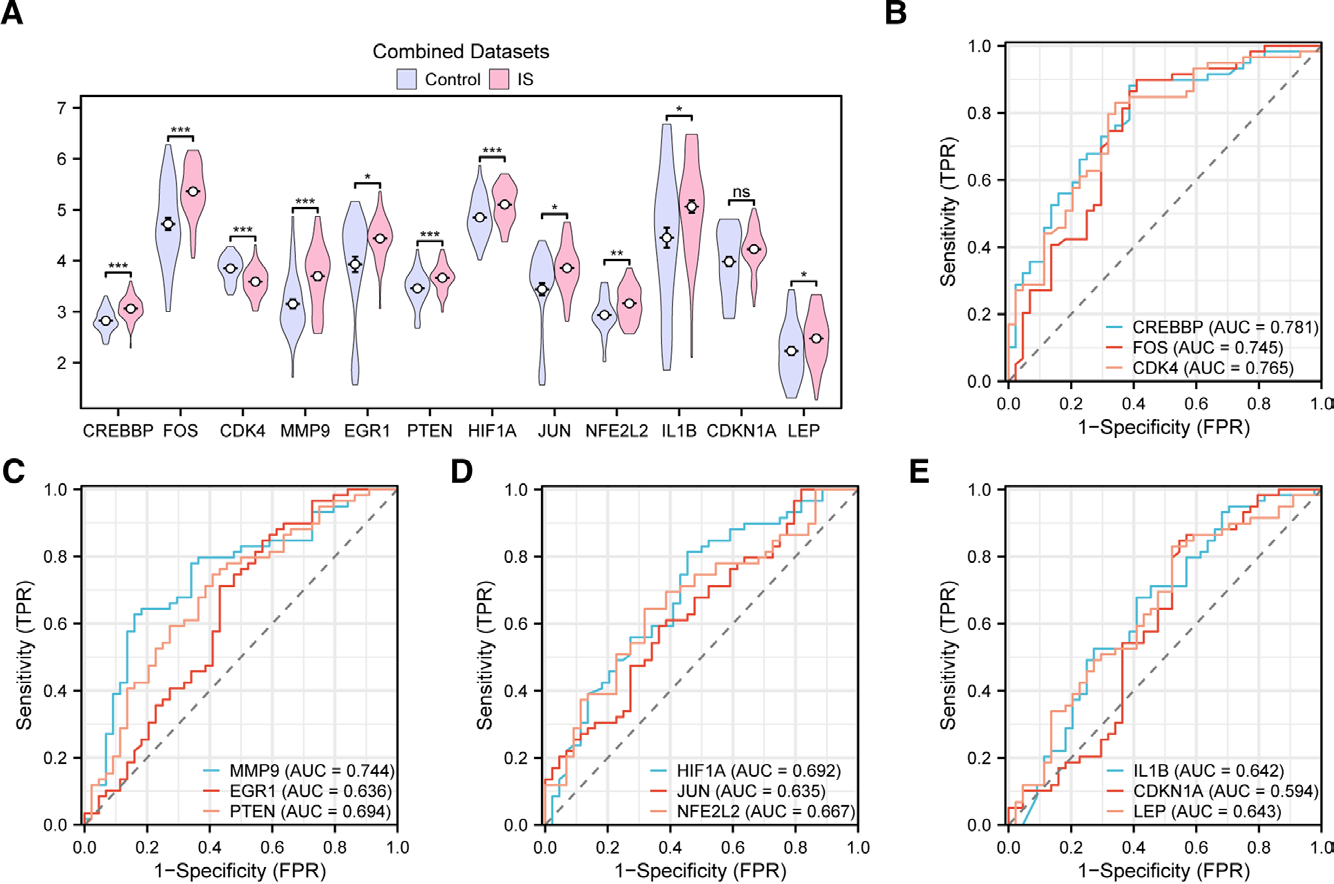
Results of differential expression validation of Hub Genes. The grouping comparison chart of Hub Genes in the ischemic stroke (IS) group and control group of the integrated GEO dataset (A). (B–E) Hub Genes: CREBBP, FOS, and CDK4 (B), MMP9, EGR1, and PTEN (C), HIF1A, JUN, and NFE2L2 (D), IL1β, CDKN1A, and LEP (E) in the ROC curves of the integrated GEO dataset. ns represents *P* value ≥ .05, not statistically significant; * represents *P* value < .05, statistically significant; ** represents *P* value < .01, highly statistically significant; *** represents *P* value < .001, extremely statistically significant. When AUC > 0.5, it indicates the trend of molecular expression promoting the occurrence of events, and the closer the AUC is to 1, the better the diagnostic effect. AUC between 0.5 and 0.7 indicates low accuracy, AUC between 0.7 and 0.9 indicates moderate accuracy. Light purple represents the control group, light red represents the ischemic stroke (IS) group. AUC = area under the curve, GEO = Gene Expression Omnibus.

The differential results show (Fig. 7A) that the expression levels of 6 Hub Genes in the integrated GEO dataset were highly statistically significant (*P* value < .001) between the IS group and the control group, namely: CREBBP, FOS, CDK4, MMP9, PTEN, and HIF1A. The ROC curve (Fig. 7B–E) indicates that the expression levels of CREBBP, FOS, CDK4, and MMP9 among the Hub Genes show a certain accuracy in distinguishing between the IS group and the control group (0.7 < AUC < 0.9); the expression levels of EGR1, PTEN, HIF1A, JUN, NFE2L2, IL1β, CDKN1A, and LEP show lower accuracy in distinguishing between the IS group and the control group (0.5 < AUC < 0.7).

### 3.8. Expression of Hub Genes in a model of OGD in human brain microvascular endothelial cells

**Figure 8.**
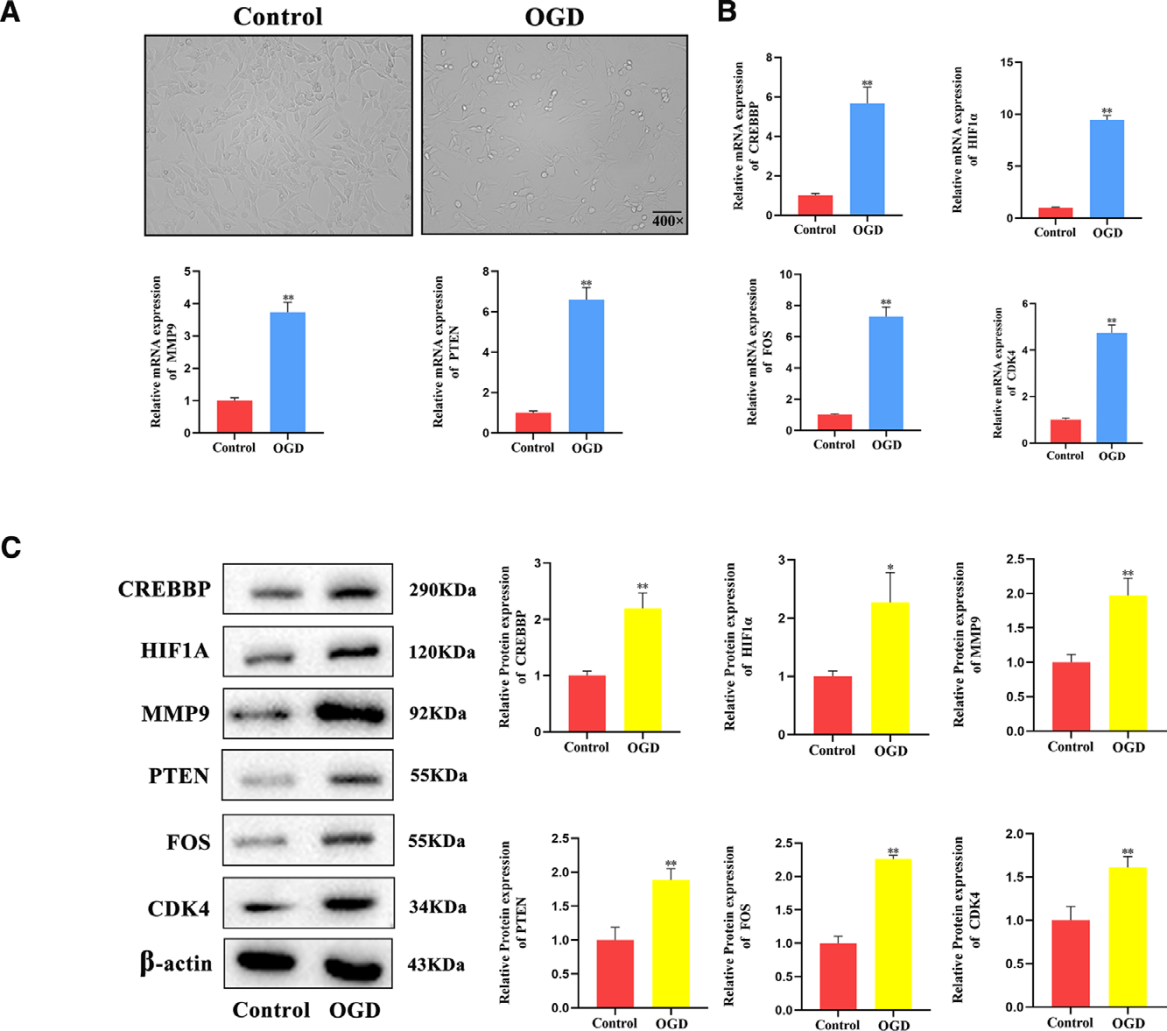
Expression of Hub Genes in a cellular oxygen-glucose deprivation model. (A) Morphological changes of human brain microvascular endothelial cells after oxygen-glucose deprivation induction (B). mRNA expression of Hub Genes in the oxygen-glucose deprivation cell model (n = 3) (C). Protein expression of hub genes in the oxygen-glucose deprivation cell model (n = 3).* represents *P* value < .05, statistically significant; ** represents *P* value < .01, highly statistically significant.

The results show that after OGD induction, human brain microvascular endothelial cells exhibit significant cell shrinkage, incomplete cell membranes, and an increased number of necrotic cells (Fig. 8A). Further detection by RT-qPCR and Western blot analysis indicates that the expression of the 6 Hub Genes CREBBP, FOS, CDK4, MMP9, PTEN, and HIF1A at the mRNA and protein levels is elevated to varying degrees in the in vitro injury model induced by OGD in human brain microvascular endothelial cells (Fig. 8B and C).

## 4. Discussion

Stroke, also referred to as a cerebrovascular accident, brain attack, or cerebrovascular insult, is a sudden disruption in the blood supply to the brain, leading to the loss of brain function. This disruption can be caused by a blockage (IS) or a rupture (hemorrhagic stroke) of a blood vessel in the brain.^[31]^ In contemporary society, the high incidence of stroke poses a significant threat to the health of the Chinese population and has become the leading cause of death and disability among Chinese residents. According to statistics, IS patients account for approximately 82.6% of all stroke cases, ranking first in stroke incidence and being the leading cause of death among stroke patients.^[32]^ Currently, intravenous thrombolysis with alteplase and mechanical thrombectomy are the primary treatment methods for IS. The earlier alteplase is administered intravenously after the onset of IS, the higher the likelihood of vascular recanalization, resulting in better improvement in the neurological deficits of stroke patients.^[33]^ In clinical practice, intravenous alteplase should ideally be administered within 3 hours, with a maximum extension to 4.5 hours. Due to the strict time window limitations for thrombolysis, the proportion of IS patients receiving intravenous thrombolysis in clinical practice is relatively low.^[34]^ Similarly, mechanical thrombectomy, another crucial treatment method for acute IS, also has a time window limitation. The time window for thrombectomy depends on the patient’s infarcted blood vessel, with the clinical consensus being that the latest time for thrombectomy should not exceed 24 hours.^[35]^ This results in high rates of death and disability from IS. Therefore, the development of more effective treatment methods for IS remains a significant research focus.

Aging is widely recognized as a major risk factor for IS.^[36]^ The fundamental mechanism of aging is cellular senescence. The enhanced SASP promotes the production of inflammatory cytokines and chemokines, increases reactive oxygen species levels, and disrupts lipid mediator synthesis, resulting in endothelial dysfunction and pathological remodeling of the extracellular matrix, which in turn contributes to atherosclerosis and arterial thrombosis.^[37,38]^ Atherosclerosis and thrombosis represent the primary etiological and pathological processes underlying IS. Studies have shown that aged vascular smooth muscle cells exacerbate chronic vascular inflammation by secreting interleukin-1α, and foam macrophages,^[39]^ one of the markers of atherosclerotic aging, increase the synthesis of metalloproteinases, leading to the degradation of elastin fibers in plaques and thinning of the fibrous cap, thereby increasing plaque instability.^[40]^ Experimental evidence indicates that chronic administration of antiaging agents alleviates atherosclerotic plaque formation in aged mice by promoting the clearance of senescent cells.^[41]^ Martin J et al reported significant lipofuscin accumulation and elevated levels of SASP-associated cytokines in the infarct region of the rat middle cerebral artery occlusion model, providing compelling experimental evidence that brain cell senescence accompanies acute cerebral ischemia.^[42]^

The molecular mechanism underlying circadian rhythms involves the periodic expression of clock genes. The central circadian clock resides in the SCN of the brain, while peripheral clocks are distributed across various tissues and organs. Under the regulation of this system, organisms exhibit ~24-hour rhythmic oscillations.^[43]^ In response to external stimuli such as light, the retina transmits signals to the SCN, which in turn generates rhythmic outputs by regulating the periodic expression of clock genes, thereby maintaining the body’s circadian oscillations.^[44]^ Growing evidence indicates that circadian rhythms play a crucial role in the pathogenesis of IS. Circadian fluctuations are observed in IS onset, with clinical studies reporting a significantly higher incidence between midnight and 6 am, and patients with nighttime onset exhibiting larger infarct volumes than those with daytime onset. Disruption of circadian rhythms exacerbates IS severity and is associated with poorer clinical outcomes.^[45–47]^ Preclinical studies have demonstrated that circadian rhythm disruption aggravates stroke severity and impairs angiogenesis in rat models of middle cerebral artery occlusion.^[48]^ Further investigations into molecular mechanisms have shown that silencing circadian clock genes suppresses autophagy in endothelial cells and accelerates the development of atherosclerotic plaques,^[49]^ while mice with targeted deletion of the circadian clock gene Bmal1 display endothelial dysfunction and a hypercoagulable state, thereby increasing the risk of both arterial and venous thrombosis.^[50,51]^ These pathological changes collectively elevate the risk of IS. Melatonin, a neurohormone synthesized in the pineal gland, regulates sleep and circadian rhythms,^[52,53]^ and experimental studies have demonstrated that melatonin mitigates ischemia-reperfusion injury in diabetic mice by enhancing autophagy via the SIRT1–Bmal1 signaling pathway.^[54]^ IS is accompanied by neuroinflammatory responses, microglia are the main immune cells of the CNS, and biological clock genes can regulate the expression of inflammatory factors in microglia.^[55]^ Further research on whether regulation of biological clock genes can mitigate neuroinflammatory responses after IS and improve the prognosis of patients with IS is warranted.

This study employed bioinformatics approaches to analyze genes associated with cellular senescence and circadian rhythms in IS. A total of 717 DEGs were identified from IS-related GEO datasets, including 380 upregulated and 337 downregulated genes. Among these, 17 genes were identified as being associated with cellular senescence and circadian rhythm-related differential expression, including CREBBP, FOS, CDK4, MMP9, MIF, ETV6, EGR1, PTEN, HIF1A, JUN, NFE2L2, OGT, BLK, RNASEL, IL1β, CDKN1A, and LEP. GO and KEGG enrichment analyses indicated that these 17 DEGs were primarily involved in biological responses such as hypoxia, cellular responses to environmental stimuli, human T-cell leukemia virus 1 infection, fluid shear stress, and atherosclerosis. GSEA further revealed that genes in the integrated GEO dataset were significantly enriched in inflammation-related biological functions and signaling pathways, including neutrophil degranulation and interleukin-4 and interleukin-13 signaling. These enrichment results highlight the specific biological mechanisms by which inflammatory gene pathways contribute to the pathogenesis of IS. Further analysis of the PPI network identified 12 Hub Genes associated with IS, including CREBBP, FOS, CDK4, MMP9, EGR1, PTEN, HIF1A, JUN, NFE2L2, IL1B, CDKN1A, and LEP. Immuno-infiltration analysis revealed that various immune cells, including activated B cells, activated CD8 T cells, and central memory CD8 T cells, are closely associated with the incidence of IS. The correlation between the 12 Hub Genes and various immune cells was subsequently analyzed. The results revealed that CDK4, among the Hub Genes, showed the strongest positive correlation with activated CD8 T cells and the strongest negative correlation with eosinophils. Differential expression analysis confirmed that the expression levels of 6 Hub Genes were significantly different between the IS and control groups: CREBBP, FOS, CDK4, MMP9, PTEN, and HIF1A. The ROC curves demonstrated that CREBBP, FOS, CDK4, and MMP9 exhibited relatively high accuracy in distinguishing between the IS and control groups. Subsequently, we established an in vitro injury model of IS induced by OGD in human brain microvascular endothelial cells, using RT-qPCR and Western blot to detect the expression of the 6 Hub Genes: CREBBP, FOS, CDK4, MMP9, PTEN, and HIF1A. The results showed that these 6 Hub Genes exhibited varying degrees of elevation in the in vitro injury model of IS induced by OGD. This suggests that IS is closely linked to several pathophysiological processes, including the activation of brain immune cells, angiogenesis, and inflammatory responses. Inhibiting the expression of these genes post-IS may reduce mortality and disability rates and promote the repair of neural functions. These Hub Genes are crucial for understanding the regulation of cellular senescence and circadian rhythm in the complex molecular mechanisms of IS and may become new therapeutic targets.

Although this study has preliminarily screened and validated several hub genes associated with IS using bioinformatics analysis and in vitro cell experiments, certain limitations still exist. The most significant limitation is the lack of systematic validation in animal models and clinical samples from IS patients. Animal experiments play a crucial role as a bridge between basic research and clinical application, offering a realistic simulation of the pathophysiological processes of human diseases, particularly in terms of the dynamic interactions within the neurovascular unit, the integrity of the blood-brain barrier, and systemic inflammatory responses. Validating clinical samples from IS patients is essential for the translational application of research findings, as these samples accurately reflect the molecular characteristics and individual variations of human diseases. In subsequent animal experimental research, we plan to utilize a middle cerebral artery occlusion rat model to dynamically monitor the expression changes of these hub genes at various time points (such as 6h, 24h, and 72h post-ischemia) using techniques like real-time quantitative PCR, Western blot, and immunohistochemistry. We will also integrate neurological scoring, infarct volume measurement, and pathological examination to comprehensively analyze the correlation between the expression levels of these genes and the extent of neuronal injury. This approach will provide more direct experimental evidence to elucidate the molecular regulatory mechanisms of these hub genes in ischemic brain injury. In subsequent clinical sample research, we plan to collect 100 to 150 cases of acute IS patients along with matched healthy controls to assess the expression levels of these hub genes in blood and cerebrospinal fluid using real-time quantitative PCR and Western blot techniques and analyze their correlation with disease severity and prognosis. The acquisition of these animal experimental and clinical sample data will significantly enhance the credibility of the findings and provide a solid theoretical foundation for future clinical translational research.

## Author contributions

**Conceptualization:** Fukang Zeng.

**Data curation:** Fukang Zeng, Mengjuan Wang, Menghao He.

**Investigation:** Fukang Zeng.

**Methodology:** Fukang Zeng, Mengjuan Wang, Menghao He.

**Project administration:** Yuxing Zhang, Zhong Li.

**Software:** Fukang Zeng.

**Validation:** Fukang Zeng.

**Visualization:** Fukang Zeng.

**Writing – original draft:** Fukang Zeng.

**Writing – review & editing:** Yuxing Zhang, Zhong Li.
